# On the Method Employed for the Cure of the Venereal Disease, about the Middle and Latter Part of the Sixteenth Century

**Published:** 1818-11

**Authors:** 


*For the London Medical and Physical Journal.*

### *On the Method employed for the Cure of the Venereal Disease, about the middle and latter Part of the Sixteenth Century.*

Collected by EURYPHON.

Αλλα πίθεσθ' ἄμφω δὲ νεωτέρω ἐστὸν ἐμεῖο.

ON reading the account related in the last Number of your Journal of the mode of treatment employed by M. OSBECK for the cure of *Syphilis*, I was forcibly struck by its resemblance to that adopted by some of the most intelligent and experienced surgeons in Europe, before the use of mercury became general. M. Osbeck is doubtless acquainted with this circumstance, and would, I think, have acted prudently had he stated it in the account he has published of his own method, the propriety of which would appear more evident with the support of such authority. As the coincidence I have spoken of is not mentioned in the account given in your Journal, I suppose it has not been noticed by him.

The opinion generally held during the last two centuries, respecting the means of cure for the venereal disease, has caused the writings of the surgeons to whom I have alluded, to be very unjustly neglected; they have consequently become scarce, and inaccessible to the generality of students. Of the value of their observations, particularly when it is considered that they wrote so early after the supposed first-appearance of the malady in Europe, there can be but one opinion among those who have diligently perused their works. They must, indeed, be considered peculiarly interesting at the present period, when hopes of curing the disease without the use of mercury are so confidently entertained by many surgeons of eminent talents and extensive experience. In collecting, therefore, their most important opinions respecting the nature of the disease, and the principal traits of their mode of cure, I shall, I trust, perform a task not unacceptable to the profession.

I commence with FERNELIUS,—a man of great erudition, experience, and knowledge in all the branches of science connected with medicine; whose works, written in language of such purity and elegance as to be hardly inferior to that of CELSUS, were for half a century universally studied in all parts of Europe. Indeed, in the words of GISELLINUS, "the repute in which they were held, and the advantages derived from the study of them, were such, that those engaged in the practice of medicine were not satisfied unless possessed of them; and, without them, the student would consider his sources for acquiring information essentially defective."*

After a brief history of the effects of the disease, and the lamentable consequences of the use of mercury as a means of cure, Fernelius enters into the consideration of the method he had employed in an extensive practice during the space of thirty years. He endeavours to shew the different results of the treatment by mercury and the means he advises, and points out the severity of the one and the mildness of the other, with an energy of style and earnestness of manner which shew the warmth of his philanthropic feelings, and evince the truth of what he asserts; that, in publishing his opinions and the results of his experience, he is influenced by a desire to relieve the sufferings of human nature, and by the consideration that, in withholding them, he should not perform his duty as a member of civilized society.

"*The circumstances to be attended to previously to the commencement of the means of cure*," are first considered.

"Before the cure is attempted," he observes, "a knowledge of many things must be acquired; and particularly the length of time which the disease may have existed, and the symptoms which have appeared. When it is recent, and the fluids only affected, or the solids but slightly so, it is not attended with any symptoms of great importance: a few blotches generally appear on the skin, which are easily removed. When hard tumors arise, and the patient is afflicted with severe pains in different parts, the cure is more difficult; and, when the bones become carious, the cartilages of the joints ulcerated, and tubercles and ulcers appear both in the viscera and the external parts of the body, as I have observed in dissections of those who have died in this state, the utmost care and attention are requisite to effect the cure. We must also consider the constitution of the patient, and the particular condition of the different organs. For a person of a spare habit, with pallid countenance, and whose stomach is weak, requires a more careful and gentle mode of treatment than persons of a full phlegmatic habit; as do the latter different means from those employed for the robust

---

* *In Prefatione Operum Fernelii,* sub finem.—BRUGIS 1579.

and vigorous. We must also act cautiously in the treatment of persons who are affected with diseased liver, and a deranged state of the functions of the stomach from an intemperate mode of life. These patients will neither bear great abstinence from food, copious long-continued sweating, nor active purgatives, without suffering serious and distressing disorder of the stomach. The state of the primæ viæ must also be attended to. If disease exist in any part, or an unwholesome diet has been long used; or the intestines are loaded with collections of feculent matter; or the lacteal vessels *(primæ venæ)* obstructed; the cure of the disease should not be attempted until those derangements are removed."—"The season of the year, the climate, and the state of the air, must also be considered; as the means employed must be more mild during a hot than during a cool temperature of the atmosphere."

Rules for three different modes of treatment are delivered by Fernelius, as applicable to different stages of the disease; which I shall consider in the order in which he has arranged them. He first speaks of a method, which he calls *imperfect.*

"The danger and inefficacy of the treatment with mercurial inunction, of which I have already spoken, induced our predecessors to make further attempts by other means. They commenced with the exhibition of active purgatives; after which, in those who could bear it, blood-letting was employed; this was followed by the use of a variety of syrups, &c. for a certain time, when the course of purgative medicines was repeated. These measures, in recent cases, and when only the fluids were contaminated, were certainly often effectual; but, if the solids had become affected, they merely relieved the present symptoms of the disease, which re-appeared at some future period with increased severity, —the malady making further progress unobserved, like a tree extending its roots deeper into the earth, and spreading them in all directions; thus acquiring a firmness of hold, which our utmost efforts can hardly destroy. These measures, therefore, removed the effects of the disease, not its cause: they deferred the evil consequences, but did not prevent them.

"Another method, founded nearly on the same principles as the preceding, was next adopted. It consisted in the use of a light and extremely spare diet, and the decoction of guaiacum, or china-root; by which the body became much extenuated, the fluids being expelled by sweat and urine. The treatment with guaiacum is certainly more safe and more efficacious than the former one, but it by no means ensures the patient from the danger of a relapse, particularly when the disease has been of long continuance, or the solids have become affected."

The cure by *regulated diet*, sweating, the exhibition of *guaiacum*, and the preparation which the author calls his *opiate*, are next particularly described. Of this I shall give an abridged account, rather than a translation.

In winter, the patient was confined in a chamber, the temperature of which was constantly kept nearly the same by fires, and great care taken to prevent the admission of currents of cold air. In summer, the patient was permitted to go into the open air during the middle of the day. The bowels were first well purged; two days after which some blood was taken from the arm. On the following day, the patient began the use of a decoction of guaiacum, made in the following manner:—Half a pound of rasped guaiacum-wood was macerated in six pints of water during twenty-four hours; the mixture was then gently boiled, until reduced to two pints. Six ounces of this were taken about five o'clock in the morning, and at six in the evening; after which the patient lay in bed, well covered with clothes, for three or four hours; if copious sweating did not ensue, attempts were made to induce it, by applying bladders and bottles filled with hot water to various parts of the body, and the head was covered with flannel. After this he took food, of which a quantity only just sufficient to support his strength was allowed. In inveterate cases it was limited to two ounces of biscuit daily, with some raisins or prunes. Delicate persons were, in general, suffered to take four ounces of bread daily; those of greater strength, six ounces. Raisins and a small quantity of biscuit were taken occasionally during the day. No animal food whatever was permitted. The common drink was a weak decoction of guaiacum. Wine conjoined with this plan, Fernelius observes, is poison. The mind of the patient was kept constantly occupied by all the agreeable amusements this state of confinement would permit; all causes of solicitude and anxiety were carefully removed. The bowels were kept open by gentle purgatives. Sometimes pains in various parts of the body, particularly about the back and loins, came on; but they soon subsided without any remission of the plan of treatment. About the twelfth day the use of the *opiate* was begun; half a drachm of which was taken after each dose of the guaiacum for eight or ten days, or until all the symptoms of the disease were removed. The patient was then permitted to go out, if the weather were at all temperate; and at this period the use of a warm bath, every evening, was commenced; on coming from which, he took five ounces of the *aqua mirifica*,* and went to bed. This plan was continued for seven or eight days; after which, the bowels were well purged for six or seven days; the weak decoction of guaiacum was used for about a fortnight longer, and the patient then gradually returned to his usual mode of life. The time generally required for the cure was about thirty days. If a relapse of any of the symptoms took place, the same plan was again adopted. Those who had been repeatedly salivated were with great difficulty cured by these means; and it was found necessary to continue the use of the decoction of guaiacum for nearly a year, in many such instances. A case of the latter kind is related by Fernelius, in a section of his work, in which he treats of the *most perfect mode of cure*. Of this I shall give an abridged translation.

When the disease has been long neglected, or numerous ineffectual attempts have been made to effect its cure, and the patient becomes afflicted with excruciating pains, and bony tumors on the head, shins, and various other parts of the body, in addition to the means above recommended, others must be resorted to for the more effectual relief of the various distressing symptoms; to exemplify which the following case is related.

M—, prior of the monastery of St. Dennis, about forty years of age, and of a full and vigorous habit of body, became affected with the venereal disease.† He immediately had a consultation of the most eminent physicians and surgeons of Paris; the determination of which was, that he should be purged, bled, and then use mercurial inunction. A copious salivation and purging came on about the seventh day: these were considered favourable circumstances, and his surgeons promised him a speedy cure. Three weeks after this, finding the symptoms not relieved, a consultation of the medical advisers was again obtained, and a repetition of the use of mercury was ordered. But, as this was not more effectual, it was directed to be carried to a still greater extent. Matters went on thus for three months, when nodes appeared on the bones of the head, arms, and thighs, accompanied with the most excruciating pain. The surgeons were again consulted, who persisted in advising the use of mercury; which was done for two years without the least benefit. He then, using the words of Fernelius, appeared more like a skeleton than a living body: he had hardly slept

---

* The prescription for the preparation of this medicine and *the opiate* I will give hereafter.

† The particular symptoms are not mentioned by Fernelius.

for two years, in consequence of the pain he suffered from the tumors of the bones. The surgeons, then finding their attempts ineffectual, had recourse to desperate measures: the bony tumors were exposed, and removed by the saw, and terebellum; and mercurial plasters were applied over the whole body for a month, and the decoction of guaiacum was administered for the same space of time. After this they entirely deserted him. Fernelius says, who would not suppose that these men were quacks or barbers? will it be believed that they were the surgeons of greatest repute in the largest city in Europe? The unfortunate prior then came under the care of our author, who proceeds to relate "*by what means he was restored.*"

He was directed a light and nourishing diet, and the decoction of guaiacum, to which an addition was made of hartshorn shavings, and some aromatic herbs—as bugloss, borage, purslane, and pimpinella; he also took six ounces of the expressed juice of those herbs twice a-day. By these means, his strength was, after a short time, much improved; when six ounces of the following decoction was ordered to be taken twice a day:—Take of rasped guaiacum-wood, ℥ij; powder of the bark of the same, ℥iſs; elecampane root, polypody of the oak, juniper berries, succory root, of each ℥i; raisins, ℥iſs; carduus benedictus, tormentil root, of each ℥ſs; pimpinella, chickweed, field-scabious, of each as much as can be taken up with the finger and thumb: to be first infused in two pints of boiling water, and then boiled to one pint.

Purgative medicines were directed to be taken once a-week.

The following was applied as a cataplasm to the tumors:—Take of mustard-seed, ℥ij, infuse for twenty hours in vinegar; then bruise them, and mix with fresh white and black briony root, reduced to a pulp, each ℥ij; hog's-lard equal in weight to the whole of the former ingredients: to be daily applied to the parts in pain.

When the patient had become relieved from pain by these means, and regained a tolerable degree of strength, he was directed to use a warm bath every third day, and to continue in it as long each time as his strength would permit. Previously to his going into the bath, he took six ounces of the *decoction* just described, with one drachm of the *opiate*. On removing from it, he was placed before a fire, and rubbed all over with an embrocation made by boiling a variety of spices and aromatic herbs in oil. He was then directed a light and spare diet. In a little more than two months, he had nearly regained his former health and strength; and he subsequently perfectly recovered, without having any symptoms of the disease recur.

Fernelius relates another case, to shew the effect of these remedies in the earlier stages of the disease.—A man came to me, he says, with a large ulcer, which had destroyed the integuments from the root of the penis and groin of one side, as far upwards as the navel: he had applied the plaster of De Vigo, with a double proportion of mercury, to the parts, and used mercurial inunction, without checking the progress of the disease. He was so much reduced by it as to be little more than skin and bone. The fauces, uvula, and soft parts of the nose, were nearly destroyed, and the ossa palati had become carious. At the end of twenty-four days, the ulcerated parts were entirely healed by the above-mentioned mode of treatment, and the local application of the *aqua divina* twice a-day.

I shall not venture to make any reflections on the nature of the above cases, but leave my readers to form their own opinions. I must, however, remark, that no further account is given by Fernelius of the origin and progress of the symptoms than what I have stated. Palmarius, the pupil of Fernelius, and who adopted the opinions of his tutor, is more particular and satisfactory in the relation of the cases he adduces to prove the efficacy of his mode of treatment. I purpose making the works of this author the subject of a future letter, should you consider the above observations worthy of being laid before your readers. I shall conclude this with an account of the mode of preparing the different remedies employed by Fernelius.

EURYPHON.

*The Alexipharmac Opiate Antidote of Fernelius.*

Take of scordium, poley-mountain *(teucrium polium*, Lin. Sp. Pl. 792. *α*), penny-royal, white horehound, origanum, mountain-mint, St. John's wort, lesser centaury, French lavender *(cassidona)*, creeping germander, common ground-pine, spikenard, of each ℥ij; anise, fennel, stone-parsley, wild carrot, rue ocymum *(ocymum basilicum*, Lin. Sp. Pl. 833), clary, *laspi (laserpitium latifolium*, Lin. Sp. Pl. 356? Laserwort), laurel-berries, and male peony seeds, of each ʒiſs; round birthwort, gentian, dittany, valerian, and asarum roots, of each ʒj; ginger, nutmeg, cloves, pepper, saffron, of each ℈iv; cinnamon, myrrh, castor, and storax, of each ʒiij; honey, a sufficient quantity to form an electuary.

*The Aqua Mirifica.*

Take of scordium, two handfuls; marsh-marigold, chickweed, pimpinella, St. John's wort, betony, marjorum, bugloss, field-scabious, and sage, of each one handful; hyssop and balm, of each a handful and a half; to be bruised, and a sufficient quantity of water poured over them, that they may be well covered by it. This mixture is to be exposed to the sun, in a close vessel, for six days; the liquor is to be expressed, and the same quantity of fresh herbs, before described, macerated in it: this is to be exposed to the sun again for ten days, the liquor expressed, and the following substances added:—tormentil, and carduus benedictus, of each ʒiſs; zedoary, nutmeg, cloves, of each ʒj; mace, ʒſs; juniper berries, ℥j; saffron, ʒj; mithridate, ℔j; treacle, ℥iij: this mixture is to be again exposed to the sun for five or six days, and then distilled. The water is to be kept in close bottles for use.

*The Aqua Divina, for Ulcers.*

Take of sublimate, twelve grains, to be dissolved in plantain-water, six ounces; and boiled in a glass vessel until half be consumed.

